# Evaluating the vertical HIV transmission risks among South African female sex workers; have we forgotten PMTCT in their HIV programming?

**DOI:** 10.1186/s12889-019-6811-4

**Published:** 2019-05-29

**Authors:** Jean Olivier Twahirwa Rwema, Stefan Baral, Sosthenes Ketende, Nancy Phaswana-Mafuya, Andrew Lambert, Zamakayise Kose, Mfezi Mcingana, Amrita Rao, Harry Hausler, Sheree Schwartz

**Affiliations:** 1 Department of Epidemiology, Key Populations Program, Center for Public Health and Human Rights, Johns Hopkins Bloomberg School of Public Health, 615 N Wolfe Street, Baltimore, MD 21205 USA; 2 The TB/HIV Care Association, Cape Town, South Africa; 3 The Human Sciences Research Council, Port Elizabeth, South Africa; 4 Nelson Mandela Metropolitan University, Port Elizabeth, South Africa; 5 The TB/HIV Care Association, Port Elizabeth, South Africa

**Keywords:** PMTCT, Vertical transmission, Female sex workers, South Africa

## Abstract

**Background:**

Female sex workers (FSW) have a greater HIV burden compared to other reproductive-aged women and experience high incidence of pregnancies. However, there are limited data on mother-to-child transmission of HIV in the context of sex work. This study assessed the uptake of prevention of mother-to-child transmission (PMTCT) services to understand the vertical HIV transmission risks among FSW in South Africa.

**Methods:**

FSW ≥18 years were recruited into a cross-sectional study using respondent-driven sampling (RDS) between October 2014–April 2015 in Port Elizabeth, South Africa. An interviewer-administered questionnaire captured information on demographics, reproductive health histories, and HIV care, including engagement in PMTCT care and ART. HIV and pregnancy testing were biologically assessed. This analysis characterizes FSW engagement in HIV prevention and treatment cascades of the four prongs of PMTCT.

**Results:**

Overall, 410 FSW were enrolled. The RDS-weighted HIV prevalence was 61.5% (95% bootstrapped confidence interval 54.1–68.0). A comprehensive assessment of the four PMTCT prongs showed gaps in cascades for each of the prongs. In Prongs 1 and 2, gaps of 42% in consistent condom use with clients among HIV-negative FSW and 43% in long-term high efficacy contraceptive method use among HIV-positive FSW were observed. The analyses for prongs three and four pertained to 192 women with children < 5 years; 101/192 knew their HIV diagnosis prior to the study, of whom 85% (86/101) had their children tested for HIV after birth, but only 36% (31/86) of those who breastfed retested their children post-breastfeeding. A substantial proportion (35%, 42/120) of all HIV-positive women with children < 5 years of age were HIV-negative at their last delivery and seroconverted after delivery. Less than half (45%) of mothers with children < 5 years (45/101) were on ART and 12% (12/101) reported at least one child under five living with HIV.

**Conclusion:**

These findings show significant gaps in engagement in the PMTCT cascades for FSW, evidenced by sub-optimal uptake of HIV prevention and treatment in the peri/post-natal periods and insufficient prevention of unintended pregnancies among FSW living with HIV. These gaps result in elevated risks for vertical transmission among FSW and the need for PMTCT services within FSW programs.

**Electronic supplementary material:**

The online version of this article (10.1186/s12889-019-6811-4) contains supplementary material, which is available to authorized users.

## Background

In 2011, the global plan towards the elimination of new HIV infections among children was launched with a target of reducing new HIV infections among children by 90% by 2015 [1]. Given that vertical mother-to-child transmission (MTCT) comprises more than 90% of pediatric HIV infections, the plan focused on the WHO’s comprehensive approach to the prevention of mother-to-child transmission of HIV (PMTCT). The four-pronged approach includes primary prevention of HIV infection among women of childbearing age, prevention of unintended pregnancies among women living with HIV, prevention of HIV transmission from women living with HIV to their children, and providing appropriate treatment, care and support to women and children living with HIV and their families [2]. The plan focused on 22 priority countries, of which 21 were from sub-Saharan Africa (SSA), and had a target to reduce vertical transmission to under 5% among breastfeeding women and to less than 2% among non-breastfeeding women [1–3].

By the end of 2015, South Africa reduced the number of new pediatric HIV infections by 84% and achieved a MTCT target rate of 2% [3]. Despite successful implementation of the PMTCT program in South Africa and ART uptake among pregnant women living with HIV surpassing 90%, neither South Africa nor other high performing PMTCT countries met the targets for reduction in overall pediatric infections [3, 4]. Analyses have highlighted that most of the PMTCT programming focus was put on prongs three and four, leaving the first two behind. For instance, there was a reduction of just 6% of new HIV infections among women of reproductive age, far below the 50% target [3]. Significant gaps also remain in the sexual and reproductive health and rights (SRHR) programming evidenced by unmet needs for family planning among women living with HIV [5]. Lastly, health disparities among specific populations, including female sex workers (FSW), result in unequal access to SRHR, HIV prevention, and HIV treatment services which may result in pockets of risk for vertical transmission for which programming efforts are currently insufficient [6].

FSW are disproportionately affected by HIV compared to other women of reproductive age in SSA [7–9]. Structural factors including stigma, discrimination, criminalization and gender-based violence put FSW at a heightened risk for HIV acquisition and transmission, while limiting FSW engagement in HIV prevention and treatment services [10, 11]. Despite the prevention and treatment needs faced by FSW, few data are available on FSW engagement in PMTCT services. FSW experience a high incidence of pregnancy and the majority are mothers; yet MTCT risks and outcomes of FSW living with HIV in SSA are largely unknown [6]. This is true in South Africa as well, where despite remarkable scale-up of the national PMTCT program, there is little knowledge of PMTCT engagement and outcomes among the FSW population.

The objective of this paper is to utilize an HIV prevention cascades framework to characterize engagement in the four prongs of the PMTCT approach among FSW in Port Elizabeth, South Africa, to assess vertical transmission risks among FSW.

## Methods

### Study design and population

Data for this analysis are from a cross-sectional study to describe HIV prevalence and MTCT risks among FSW from Port Elizabeth and the greater Nelson Mandela Bay Metropolitan Municipality (NMBM) in South Africa. FSW were recruited using respondent-driven sampling (RDS) from October 2014 to April 2015. RDS is a method to sample hidden populations like FSW by involving peers to recruit in their networks [12]. The study setting and design have been described previously [13, 14]. Briefly, nine FSW were recruited as “seeds” to recruit their peers. Each “seed” was given three referral coupons to recruit other FSW in her network into the study. Eligible participants were cisgender women who were at least 18 years old, possessing a valid study coupon, living in the NMBM and reporting sex work as their main source of income in the year preceding the study. Consideration was made in the selection of the “seeds” to ensure diversity in HIV status, race, age and location. Prior to enrolment, a short eligibility assessment was conducted by study interviewers using a structured questionnaire. Following eligibility screening, eligible women completed written informed consent in English or Xhosa prior to study enrollment. Each participant was in turn given three coupons to recruit in their network as well. Participants received reimbursement for their transport and time up to a total of US$10 for their study visit and successful recruitment of up to three FSW.

During the study, structured face-to-face interviews were conducted by trained data collectors using a standard questionnaire. Information on sociodemographic characteristics, reproductive health history, knowledge and attitudes regarding HIV and STI risk, practices with paying and nonpaying sexual partners was collected.

Rapid HIV and urine pregnancy tests were performed for all participants. HIV testing followed South African HIV testing guidelines [15]. Viral load testing was conducted at the local reference laboratory for women who were pregnant and living with HIV at the time of the study.

The study was approved by the institutional review boards of Johns Hopkins School of Public Health and the Human Sciences Research Council in South Africa.

### Statistical analyses

Descriptive statistics of participant sociodemographic and SRHR characteristics are presented as crude proportions and RDS-weighted estimates.

Engagement in PMTCT services was assessed by performing a cascade analysis for all four PMTCT prongs. PMTCT cascades have been used extensively in the literature as tools to evaluate the implementation and performance of PMTCT programs in different countries [16–18]. For prong one, primary prevention of HIV infection among women of childbearing age, we assessed consistent condom use (CCU) among HIV negative FSW by type of sexual partner, including regular and new clients, casual and long-term nonpaying partners. CCU was defined as using condoms during all their 10 most recent vaginal or anal sexual acts. Only CCU was considered for primary prevention of HIV because PrEP was recommended for FSW after data collection for this study was completed [19]. For the second prong, prevention of unintended pregnancies among women living with HIV, we assessed the use of contraception methods among FSW who were living with HIV and not trying to get pregnant at the time of study. Contraceptive use was sub-divided into two categories: use of any form of contraception, and use of more reliable long-term, non-barrier contraception methods. For the former, self-report of any of the following was assessed: condom use for family planning, birth control pill, intrauterine device (IUD), injectable birth control (Depo Provera or Nuristerate), implant (Norplant or Jadelle), diaphragm or cervical cap and tubal ligation. For long-term methods, we included the IUD, implant, injectable and tubal ligation. For prongs 3 and 4, prevention of HIV transmission from women living with HIV to their children and provision of appropriate treatment, care and support to women and children living with HIV and their families, analyses were restricted to HIV positive FSW who had children under 5 years of age to place the findings in the current PMTCT context. We used two PMTCT cascade analyses for these mothers and their infants. Prongs 3 and 4 were assessed using combined cascades, one based on engagement of mothers and the other on data regarding their children. The steps in the mothers’ cascade evaluated information on the mothers’ HIV and treatment status during pregnancy and in the years following pregnancy. Although current HIV status was biologically confirmed, HIV status during pregnancy was self-reported. The cascade for children was restricted to children born to mothers with a known HIV diagnosis prior to the study. The proportion of infants tested for HIV at least once after delivery was evaluated, along with those retested post breastfeeding and the vertical transmissions reported by mothers. All the analyses were performed with Stata Version 14.2 (StataCorp, College Station, TX).

## Results

A total of 1069 coupons were distributed by the study, of which 435 women presented to the study site. It is unknown how many of the 1069 coupons were distributed by FSW participants to other FSW. Overall, 25 did not meet the eligibility criteria and 410 including the nine seeds met the eligibility criteria and were enrolled in the study. The median number of RDS recruitment waves was 6 (IQR:4–9) while the maximum attained was 16. The median age was 28 years (IQR:19–51). Of them, 42% (172/410) were single, 84% (343/410) had been pregnant at least once and 75% (307/410) had at least one biological child. The majority, 70.6% [(243/343); RDS adjusted prevalence: 66.4%(95%CI: 58.1–74.8)], of FSW who had previously been pregnant reported a past unintended pregnancy. The crude HIV prevalence was 63.7% (95%CI: 59.0–68.3); the RDS-weighted estimate was 61.5% (95% bootstrapped confidence interval 54.1–68.0). Crude and RDS-adjusted estimates of socio-demographic and other characteristics are summarized in (Table 1).

**Table 1:** Sociodemographic and reproductive health characteristics of participating female sex workers in Port Elizabeth, South Africa, 2014–2015

Characteristic	N	Crude %	RDS-Adjusted % [95% CI]
Age			
18–24	122	29.8	38.2 (30.1–46.3)
25–34	205	50	44.0 (37.0–51.0)
> 35	83	20.2	17.8 (12.2–23.5)
Race			
Black African	337	83.2	74.5 (60.6–88.4)
Other	68	16.8	25.5 (11.6–39.4)
Education			
0 - 8th grade	87	21.2	22.9 (17.2–28.5)
Some secondary school	158	38.5	36.5 (30.5–42.5)
Secondary level or above	165	40.3	40.6 (33.9–47.3)
Relationship status			
Single	172	42.0	39.7 (32.9–46.4)
In relationship with steady partner	238	58.0	60.3 (53.6–67.1)
History of pregnancy			
Never been pregnant	67	16.3	15.9 (11.7–20.3)
Has been pregnant at least once	343	83.7	84.1 (79.7–88.3)
History of unintended pregnancy among FSW with a history of pregnancy			
Never	100	29.3	33.5 (25.2–41.9)
Has had at least one unintended pregnancy	243	70.6	66.4 (58.1–74.8)
Biological children			
Has no child	103	25.1	24.8 (19.3–30.4)
Has at least one child	307	74.9	75.2 (69.6–80.7)
Average income per week			
Less than 500 ZAR	151	36.8	37.1 (30.1–43.3)
Over 500 ZAR	259	63.2	62.9 (56.7–69.3)
Occupation			
Sex work only	391	95.4	93.7 (89.7–97.7)
Additional occupation	19	4.6	6.3 (2.3–10.2)
STI symptoms in the last year			
No	255	62.2	63.4 (56.4–70.5)
yes	155	37.8	36.6 (30.1–43.2)
HIV infection			
Negative	149	36.3	38.5 (31.2–45.8)
Positive	261	63.7	61.5 (54.2–68.8)

Regarding engagement in ANC and delivery services, 95% (183/192) of FSW reported at least one ANC visit and 94% (181/192) had been offered HIV testing services during ANC. Twenty-eight percent (54/192) of FSW were asked by a provider to bring a partner during their ANC visit and 2 of them reported to have ever been denied ANC services because their male partners had not attended. All FSW (100%) reported to have delivered at a healthcare facility.

Regarding sex work dynamics during pregnancy, among mothers engaged in sex work prior to pregnancy, women reported continuing sex work for a median of 5 months IQR [4-7] during pregnancy following pregnancy diagnosis. When considering return to sex work following pregnancy, 27% (38/143) had resumed sex work within the first 3 months after delivery, increasing to 48% (69/143) by sixth months post-partum.

### Engagement in the PMTCT cascade

**Fig. 1:**
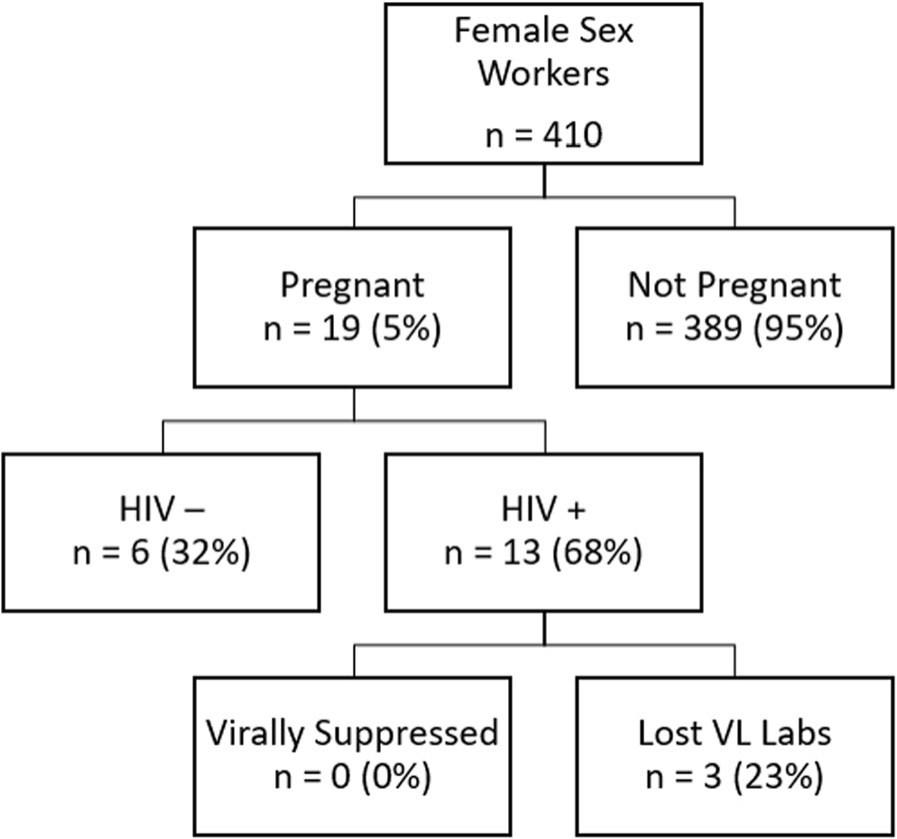
Pregnancy and HIV status at the time of the study among female sex workers in Port Elizabeth, South Africa, 2014–2015

**Fig. 2:**
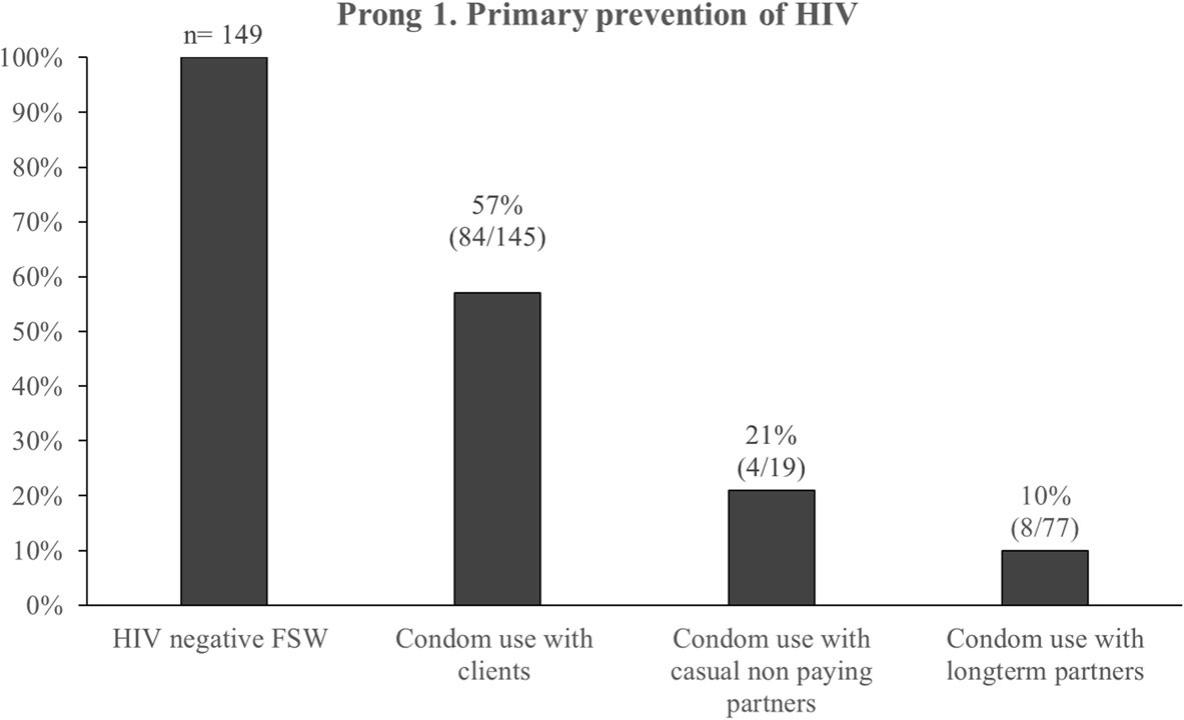
Consistent condom use in the last 10 sexual acts among HIV negative female sex workers in Port Elizabeth, South Africa, 2014–2015

At the time of the study, 5% (19/410) of all FSW were pregnant. Among pregnant FSW, 68% (13/19) were HIV positive, 31% (4/13) of whom were on ART. Viral load assessments among HIV positive pregnant women indicated that none of the pregnant FSW living with HIV were virally suppressed (Fig. 1). In terms of engagement in the four prongs of the PMTCT approach, primary prevention of HIV among women of childbearing age (Prong 1) was assessed. Among HIV negative FSW, CCU in the last 10 sexual acts was 58% (84/145) with paying clients, 21% (4/19) with casual nonpaying partners and 10% (8/77) with long-term partners (Fig. 2).

**Fig. 3:**
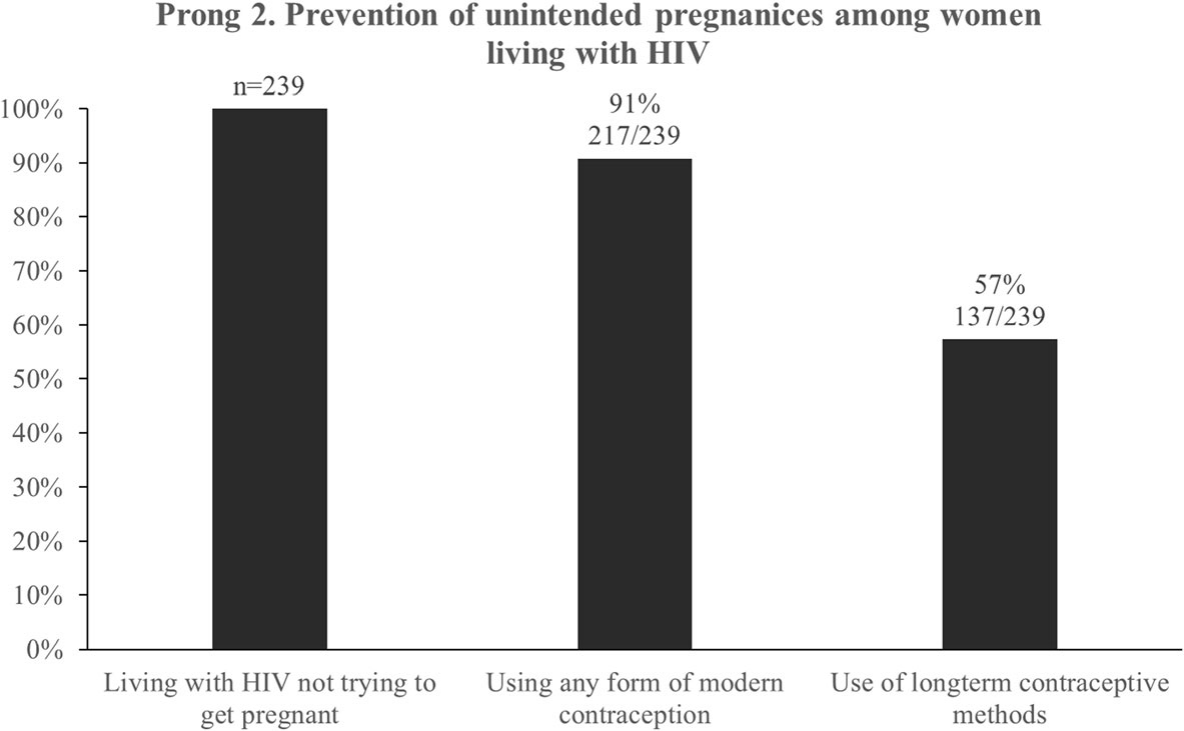
Modern contraceptive use among FSW living with HIV in Port Elizabeth, South Africa, 2014–2015

The second prong considered prevention of unintended pregnancies among women living with HIV. Of the 261 HIV positive women in the study, 22 were trying to become pregnant at the time of the study and were excluded from the prong 2 analysis. Among the remaining 239 FSW, 91% (217/239) reported using at least one contraception method. Utilization dropped to 57% (137/239) for long-term, high efficacy contraceptive methods, (Fig. 3).

**Fig. 4:**
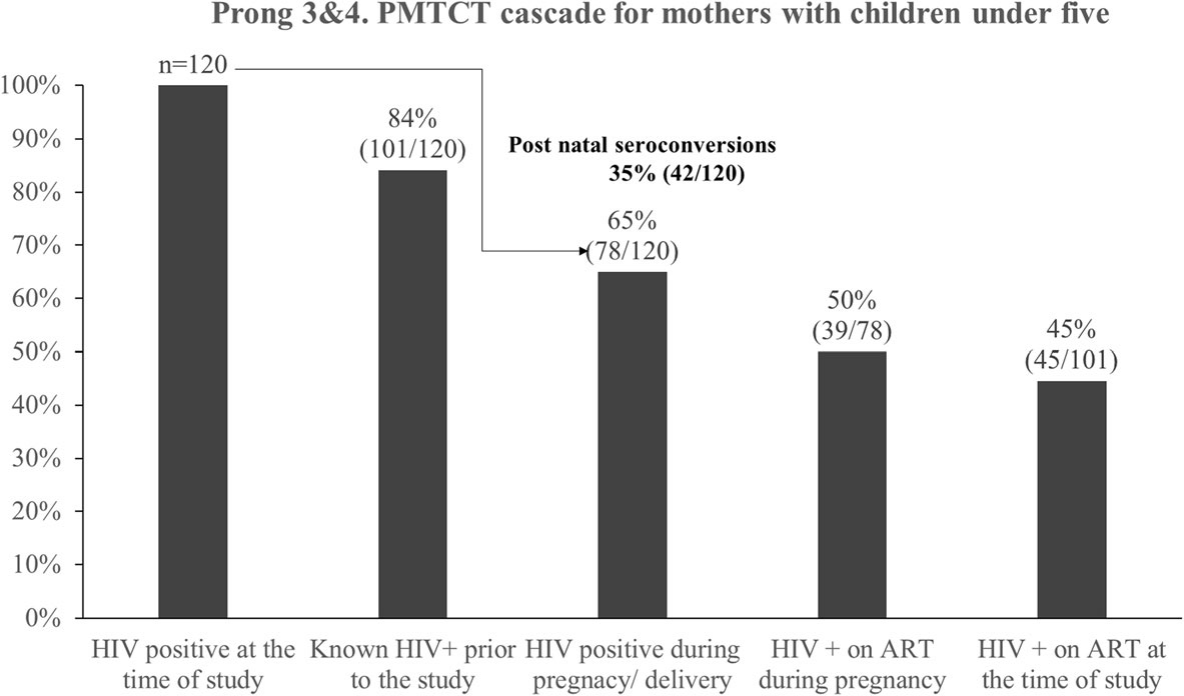
PMTCT cascade for HIV positive FSW for mothers with children under five in Port Elizabeth, South Africa, 2014–2015

When considering engagement in treatment among FSW mothers living with HIV with children under 5 years, and child testing and treatment outcomes (Prongs 3 and 4), 192 women had children under five. From the HIV testing performed by the study, 63% (120/192) of mothers were found to be HIV positive. The majority (101/120, 84%) of mothers were aware of their HIV positive status prior to the study. Among the HIV positive mothers with children under five, 65% (*n* = 78/120) were HIV positive at the time of their last delivery, while 35% (42/120) of the HIV positive mothers acquired the infection in the post-natal period. Of those who were living with HIV during pregnancy, 50% (39/78) had started ART by the time of delivery. Overall, among all mothers living with HIV with children under five, 45% (45/101) were on ART (Fig. 4).

**Fig. 5:**
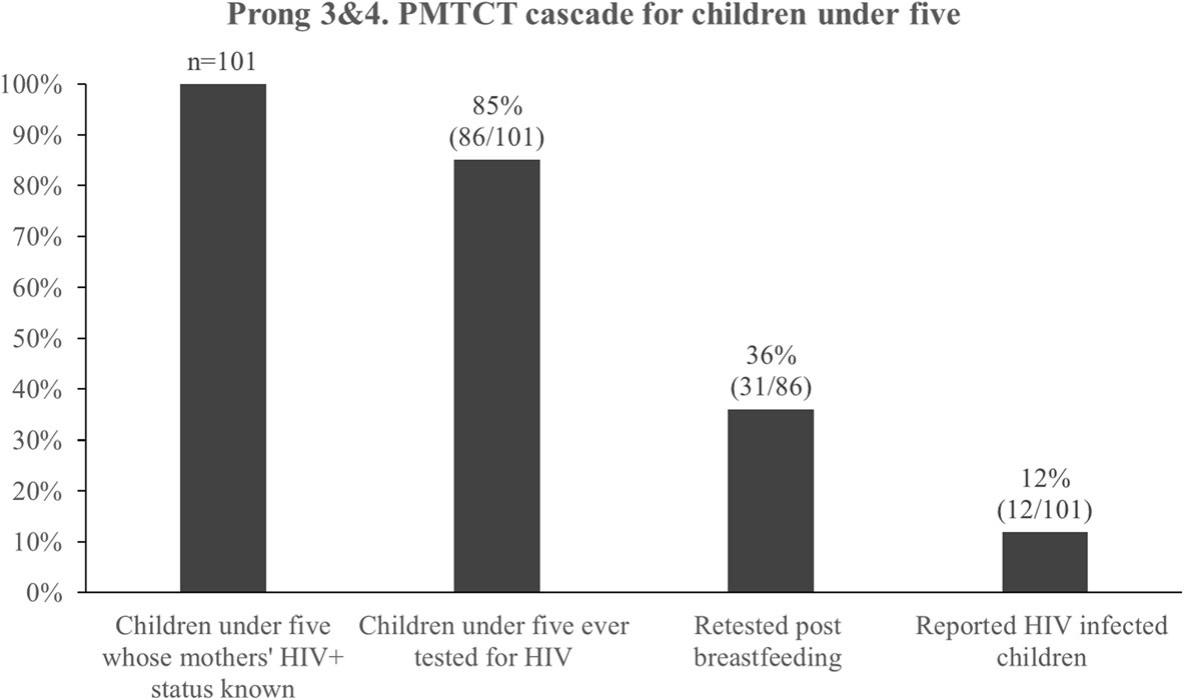
PMTCT cascade for children under five born to HIV positive FSW in Port Elizabeth, South Africa, 2014–2015

Of the 101 women who were known to be living with HIV prior to the study, 85% (86/101) had their children tested for HIV at least once after birth. The majority (85%, *n* = 86) of mothers breastfed, of which 36% (31/86) had their children retested after breastfeeding cessation. Overall, 12% (12/101) of previously diagnosed mothers reported at least one child under five living with HIV (Fig. 5).

## Discussion

Taken together these data provide evidence of the suboptimal engagement of FSW in the PMTCT care continuum that is evidenced by significant gaps in each of the four prongs of the WHO recommended PMTCT approach. Notable gaps observed included inconsistent condom use with both clients and non-paying partners among HIV negative FSW, low uptake of more reliable contraceptive methods among HIV positive FSW, insufficient ART coverage among FSW mothers living with HIV, and low retesting rates after breastfeeding. Vertical transmission risks were further evidenced from the infections reported by mothers, as 12% reported at least one child living with HIV.

The high levels of HIV acquisition among mothers in the months and years after delivery, 35% of observed infections occurred among recent mothers, provides additional evidence of the need to enhance HIV prevention efforts throughout the breastfeeding period and beyond. Protecting both the mother and the child during this period are critical given that nearly 50% of FSW resumed sex work within the 6 months after delivery. The high risk of HIV acquisition in the post-partum years highlights the need for improved attention to Prong one of the PMTCT approach and expanded primary prevention efforts for new mothers, including promotion of consistent condom use but also pre-exposure prophylaxis (PrEP). Furthermore, the South African PMTCT guidelines propose repeat HIV testing for HIV negative women during pregnancy and in the post-partum period [4]. Given the frequent postnatal HIV seroconversions among FSW, the elevated MTCT risks associated with seroconversion during pregnancy [20], promoting repeat testing among pregnant and breastfeeding FSW may be particularly important to allow for early detection of new HIV infections and early initiation of treatment [21]. PrEP use for pregnant and breastfeeding FSW could also be encouraged, given favorable safety findings to date and that is a current WHO recommendation [22–24].

Although any contraceptive use among FSW living with HIV was high, just over half of FSW reported use of long-term non-barrier contraceptive methods. This is critical given the high proportion of unintended pregnancies reported among FSW in this and prior studies [25–27]. Similar patterns were found among FSW in India, Kenya, Mozambique and South Africa [28]. Further efforts to improve more reliable contraceptive use among FSW are necessary given the high rates of unplanned pregnancies and the associated adverse outcomes including late ANC consultation and low birth weight of children [29–31]. Additionally, unintended pregnancies among FSW are also associated with social and economic consequences, including decreased ability to work and loss of income [26].

A key contribution of this work is to provide an understanding of vertical transmission risks among FSW living with HIV. ART uptake during pregnancy and the post-partum period was a critical gap observed among FSW mothers. Only 50% of FSW living with HIV started ART during pregnancy and less than half of FSW with children under five were on ART at the time of the study. In comparison, overall uptake of ART treatment among all pregnant women living with HIV in South Africa is 90% [3]. The low ART coverage among FSW has been observed in other studies, however, these data further showcase the inadequacy of routine PMTCT services for FSW. In fact, nearly all FSW with children under five attended ANC services, were offered HIV testing services during their pregnancy, but were not linked to care and treatment services. This demonstrates that despite successful PMTCT scale up in South Africa, health disparities exist in PMTCT coverage among FSW. It also provides further evidence that programs that are successful for other women of reproductive age do not necessarily work for FSW due mainly to structural factors particular to FSW that limit their ability to access HIV prevention and treatment services [11]. Discriminatory practices may also play a role here, as other studies have found that insistence upon male partner attendance in ANC is one way of excluding FSW from care [32]. Additionally, among the few FSW who were pregnant and on ART at the time of study, none were virally suppressed indicating that even those on treatment may still have an elevated risk of MTCT [33, 34]. Specific programming efforts to improve ART coverage and viral load suppression tailored to FSW mothers are necessary.

Transmission risks among FSW may be higher than the national average given that 12% of the FSW who were known to be living with HIV prior to the study reported having at least one child living with HIV. Moreover, given that the reported retesting after breastfeeding is only 30%, further undiagnosed MTCT cases likely exist.

The peer-led national sex worker strategy in South African provides a unique opportunity to address gaps in the PMTCT cascade for FSW [19]. Peer educators embedded within community-based outreach teams and central drop-in centers can help programs to identify FSW who are pregnant and support their early attendance in ANC services, support adherence of those on ART, support HIV testing services for children, find mother/infant pairs lost to follow up and support mothers in the treatment of infected children.

This study has limitations. First, the analysis relies on self-reported information for ART use, prior HIV diagnosis and children’s HIV status, which are all subject to recall and social desirability bias. However, the high correlation of self-reported HIV diagnosis and testing results increases confidence in the accuracy of the self-report. Furthermore, there is little reason to believe that the HIV infection status of the children would be overreported, however underreporting is possible, highlighting the importance for programmatic and research data that includes HIV testing of children of FSW. Restricting the analyses to women with children under five provided the possibility of placing the findings in the current PMTCT context but resulted in a smaller sample size that prevented the performance of more complex analyses. Lastly, the cross-sectional design limited our ability to assess temporal relationships, particularly of vertical transmission. Despite these limitations, clear MTCT risks were identified among the sample of mothers.

## Conclusion

This study is one of the few to assess the level of engagement in the PMTCT cascade and to evaluate vertical transmission risks among FSW in South Africa and sub-Saharan Africa. The findings demonstrate that despite the successful implementation of PMTCT programs and tremendous achievement in the reduction of vertical HIV transmission in South Africa, FSW may experience poorer access and uptake of PMTCT services compared to other women of reproductive age. FSW have high HIV acquisition risks, high unmet needs in family planning, lower ART uptake during pregnancy and in post-natal periods and may be experiencing higher MTCT rates compared to the national average. Reinforcement of specific PMTCT programming for FSW is critical to improve the health outcomes for FSW and their children.

A French translation of this article has been included as Additional file 1.

A Portuguese translation of the abstract has been included as Additional file 2.

## Additional files

Additional file 1: Translation of this article into French. (PDF 469 kb)

Additional file 2: Translation of the abstract of this article into Portuguese. (PDF 107 kb)

## Abbreviations

ANC: Antenatal care
ART: Antiretroviral treatment
CCU: Consistent Condom Use
FSW: Female Sex Workers
HIV: Human Immunodeficiency virus
IQR: Interquartile range
IUD: Intrauterine Device
MTCT: Mother-to-child Transmission of HIV
NMBM: Nelson Mandela Bay Metropolitan Municipality
PMTCT: Prevention of Mother-to-Child Transmission of HIV
PrEP: Pre-exposure Prophylaxis
RDS: Respondent Driven Sampling
SRHR: Sexual Reproductive Health and Rights
SSA: Sub-Saharan Africa
STI: Sexually Transmitted Infection
WHO: World Health Organization
