# Supplementary material for: Evaluating the vertical HIV transmission risks among South African female sex workers; have we forgotten PMTCT in their HIV programming?
Source: BMC Public Health. 2019 May 29;19(Suppl 1):605. doi: 10.1186/s12889-019-6811-4 (PMC6538543; doi:10.1186/s12889-019-6811-4)
Supplement: Supplementary file 1 — Translation of this article into French. (PDF 469 kb) [file 12889_2019_6811_MOESM1_ESM.pdf]

Évaluation des risques de transmission verticale du VIH et des cascades de soins PTME chez les travailleuses du sexe Sud-africaines. Avons-nous oublié la PTME dans leur programme de lutte contre le VIH ?

Auteurs : Jean Olivier Twahirwa Rwema (First Author), [1] Stefan Baral, [1] Sosthenes Ketende, [1] Nancy Phaswana-Mafuya,[3,4] Andrew Lambert, [2] Zamakayise Khose,[3] Mfezi Mcingana, [5] Amrita Rao [1], Harry Hausler, [2] Sheree Schwartz

Affiliations :

1. Department of Epidemiology, Center for Public Health and Human Rights, Johns Hopkins Bloomberg School of Public Health, Baltimore, Maryland, USA
2. The TB/HIV Care Association, Cape Town, South Africa
3. The Human Sciences Research Council, Port Elizabeth, South Africa
4. Nelson Mandela Metropolitan University, Port Elizabeth, South Africa
5. The TB/HIV Care Association, Port Elizabeth, South Africa

Adresses : Jean Olivier Twahirwa Rwema, MD, MPH, Department of Epidemiology, Key Populations Program, Center for Public Health and Human Rights, Johns Hopkins Bloomberg School of Public Health, 615 N Wolfe Street E 7133 Baltimore, MD 21205 (email: jtwahir1@jhu.edu)

Adresses électroniques des auteurs :

Jean Olivier Twahirwa Rwema: jtwahir1@jhmi.edu
Stefan Baral: sbaral@jhu.edu
Sosthenes Ketende: sketende@jhu.edu
Nancy Phaswana-Mafuya: nphaswanamafuya@hsrc.ac.za
Andrew Lambert: lambertandy@gmail.com
Zamakayise Khose: zkose@hsrc.ac.za
Mfezi Mcingana: mfezi@tbhivcare.org
Amrita Rao: arao24@jhu.edu
Harry Hausler: hhausler@tbhivcare.org
Sheree Schwartz: sschwartz@jhu.edu

Résumé

Contexte : Le fardeau du VIH est plus lourd pour les travailleuses du sexe (TDS) que pour les autres femmes en âge de procréer et ce groupe présente une incidence élevée de grossesses. Cependant, les données sur la transmission du VIH de la mère à l'enfant dans l'industrie du sexe sont limitées. Cette étude évaluait le recours à des services de prévention de la transmission mère-enfant (PTME) dans le but de comprendre les risques de transmission verticale du VIH parmi les TDS en Afrique du Sud.

Méthodes : Des TDS âgées d'au moins 18 ans ont été intégrées à une étude transversale utilisant un échantillonnage fondé sur les répondants (EFR) entre octobre 2014 et avril 2015 à Port Elizabeth, en Afrique du Sud. Un questionnaire administré par un enquêteur a permis de recueillir des informations sur les données démographiques, les antécédents en matière de santé reproductive et les traitements du VIH, notamment la participation à un programme de PTME et la prise d'un TAR. Des tests biologiques de grossesse et de dépistage du VIH ont été réalisés. Cette analyse évalue l'implication des TDS en matière de prévention du VIH et de cascades de soins dans les quatre composants de la PTME.

Résultats : 410 TDS au total ont été recrutées. La prévalence du VIH pondérée par l'EFR était de 61,5 % (intervalle de confiance bootstrap à 95 % 54,1-68,0). Une évaluation complète des quatre composants de la PTME a révélé un manquement à certains éléments dans les cascades de soins pour chacun de ces composants. Dans les composants 1 et 2, il a été observé que 42 % des TDS séronégatives pour le VIH n'utilisaient pas systématiquement des préservatifs avec leurs clients et que 43 % des TDS séropositives pour le VIH n'utilisaient pas de méthode de contraception très efficace à long terme. Les analyses concernant le troisième et le quatrième composant concernaient 192 femmes ayant des enfants de moins de 5 ans ;
101/192 connaissaient leur sérologie VIH avant l'étude, et 85 % d'entre elles (86/101) avaient fait dépister leurs enfants pour le VIH après leur naissance, mais seulement 36 % (31/86) de celles qui avaient allaité avaient refait faire un dépistage pour leurs enfants après l'allaitement. Une proportion non négligeable (35 %, 42/120) de toutes les femmes séropositives pour le VIH ayant des enfants de moins de 5 ans étaient séronégatives avant leur dernier accouchement et étaient devenues séropositives après l'accouchement. Moins de la moitié (45 %) des femmes

ayant des enfants de moins de 5 ans (45/101) étaient sous TAR et 12 % (12/101) avaient déclaré avoir au moins un enfant de moins de 5 ans vivant avec le VIH.

Conclusion : Ces résultats montrent des manquements significatifs dans l'implication des TDS dans les cascades de PTME, mis en évidence par une utilisation sous-optimale de la prévention et du traitement contre le VIH dans la période prénatale et postnatale ainsi que par une prévention insuffisante des grossesses non désirées chez les TDS vivant avec le VIH. Ces manquements entrainent des risques élevés de transmission verticale chez les TDS et soulignent la nécessité d'intégrer des services de PTME dans les programmes destinés aux TDS.

Mots clés : *PTME, transmission verticale, travailleuses du sexe, Afrique du Sud*

Contexte

En 2011 était lancé le plan mondial pour l'élimination des nouvelles infections au VIH chez les enfants, avec pour objectif de réduire de 90 % les nouvelles infections au VIH chez les enfants d'ici 2015.[1] Dans la mesure où la transmission verticale de la mère à l'enfant (TME) représente plus de 90 % des infections pédiatriques par le VIH, le plan était axé sur l'approche complète de l'OMS pour la prévention de la transmission de la mère à l'enfant (PTME). Cette approche, constituée de quatre composants, comprend une prévention primaire des infections par le VIH chez les femmes en âge de procréer, la prévention des grossesses non désirées chez les femmes vivant avec le VIH, la prévention de la transmission des mères vivant avec le VIH à leurs enfants et l'administration de soins, de traitements et d'un soutien appropriés aux femmes et aux enfants vivant avec le VIH et à leurs familles.[2] Le plan était axé sur 22 pays prioritaires, dont 21 en Afrique subsaharienne (ASS), et avait pour objectif de réduire la transmission verticale

pour qu'elle atteigne moins de 5 % chez les femmes allaitantes et moins de 2 % chez les femmes non allaitantes.[1-3]

Fin 2015, l'Afrique du Sud avait réduit le nombre de nouvelles infections pédiatriques au VIH de 84 % et avait atteint l'objectif de TME de 2 %.[3] Cependant, malgré la mise en oeuvre réussie du programme PTME en Afrique du Sud et l'administration d'un TAR chez 90 % des femmes enceintes vivant avec le VIH, ni l'Afrique du Sud ni aucun autre pays pratiquant une PTME très efficace n'avait atteint l'objectif de réduction du nombre total d'infections pédiatriques.[3, 4] Les analyses ont mis en évidence que les programmes de PTME visaient surtout les composants trois et quatre, au détriment des deux premiers. Par exemple, le nombre de nouvelles infections au VIH chez les femmes en âge de procréer n'avait été réduit que de 6 %, bien loin des 50 % visés.[3] Il reste également des manques importants dans le programme de santé et droits sexuels et reproductifs (SDSR), mis en évidence par des besoins de planification familiale non pris en charge pour les femmes vivant avec le VIH.[5] Enfin, les disparités de santé dans des populations spécifiques, notamment les travailleuses du sexe (TDS), entraînent un accès inégal aux SDSR, à la prévention du VIH et aux traitements du VIH, qui peuvent créer des poches de risques de transmission verticale pour lesquelles les efforts produits par le programme sont actuellement insuffisants.[6]

En Afrique subsaharienne, les TDS sont touchées par le VIH de manière disproportionnée par rapport aux autres femmes en âge de procréer.[7-9] Des facteurs structurels, notamment la stigmatisation, la discrimination, l'incrimination et les violences sexuelles, augmentent le risque d'acquisition et de transmission du VIH chez les TDS, tout en limitant leur implication dans les services de prévention et de traitement du VIH.[10, 11] Par ailleurs, malgré les besoins de

prévention et de traitement des TDS, peu de données sont disponibles sur leur implication dans les services de PTME. Les TDS présentent une incidence élevée de grossesse et la majorité d'entre elles ont des enfants ; cependant, les risques et les survenues de TME chez les TDS vivant avec le VIH sont très mal connus en Afrique subsaharienne.[6] Cela est également vrai en Afrique du Sud où, malgré une avancée remarquable du programme de PTME national, l'implication des TDS et les données les concernant sont très peu connues.

L'objectif de ce document est d'utiliser une structure en cascades pour la prévention du VIH afin de définir l'implication des TDS envers les quatre composants de la PTME à Port Elizabeth, en Afrique du Sud, et ainsi évaluer les risques de transmission verticale chez les TDS.

Méthodes

Conception de l'étude et population

Les données utilisées pour cette analyse proviennent d'une étude transversale visant à décrire la prévalence du VIH et les risques de TME chez les TDS de Port Elizabeth et dans la zone plus importante de la métropole de la baie Nelson Mandela (Nelson Mandela Bay Metropolitan Municipality, NMBM) en Afrique du Sud. Les TDS ont été recrutées via un échantillonnage en fonction des répondants (EFR) entre octobre 2014 et avril 2015. L'EFR est une méthode d'échantillonnage des populations cachées, comme les TDS, consistant à impliquer les pairs pour qu'ils recrutent des participants dans leur secteur géographique.[12] La zone géographique de l'étude et sa conception ont été décrites précédemment.[13, 14] Pour résumer, neuf TDS ont été recrutées en tant que « rabatteuses » pour recruter leurs pairs. Chaque « rabatteuse » a reçu trois coupons correspondant à d'autres TDS à recruter dans son

secteur de l'étude. Les participantes éligibles étaient des femmes cisgenres âgées d'au moins 18 ans, possédant un coupon de l'étude valable, vivant dans la NMBM et ayant déclaré avoir eu comme principale source de revenus un travail dans l'industrie du sexe durant l'année précédant l'étude. Le choix des « rabatteuses » a été fait de manière à garantir une diversité dans la sérologie VIH, la race, l'âge et la zone géographique. Avant le recrutement, les enquêteurs de l'étude ont évalué brièvement l'éligibilité des participantes potentielles à l'aide d'un questionnaire structuré. Après vérification de leur éligibilité, les femmes éligibles ont rempli un formulaire de consentement éclairé, en anglais ou en xhosa, avant d'être recrutées dans l'étude. Chaque participante a ensuite reçu trois coupons pour recruter à son tour des femmes dans son secteur géographique. Les participantes ont été remboursées de leurs frais de transport et de leur temps passé jusqu'à un total de 10 USD pour leur visite de l'étude et le recrutement effectif de 1 à 3 TDS.

Durant l'étude, des entretiens structurés en tête à tête ont été menés par des personnes formées chargées de collecter les données à l'aide d'un questionnaire standard. Des informations ont également été recueillies sur les caractéristiques sociodémographiques, les antécédents de santé reproductive, les connaissances et les attitudes relatives au VIH et au risque d'IST, ainsi que sur les pratiques avec des partenaires payants et non payants.

Des tests de dépistage du VIH et des tests de grossesse urinaires ont été rapidement effectués pour toutes les participantes. Les tests de dépistage du VIH étaient conformes aux directives Sud-africaines pour le dépistage du VIH.[15]Des tests de mesure de la charge virale ont été effectués dans le laboratoire de référence local pour les femmes enceintes vivant avec le VIH au moment de l'étude.

L'étude a été approuvée par les comités d'examens institutionnels de la Johns Hopkins Bloomberg School of Public Health et du Human Sciences Research Council of South Africa.

Analyses statistiques

Les statistiques descriptives des caractéristiques sociodémographiques et SDSR des participantes sont présentées sous forme de proportions brutes et d'estimations pondérées par l'EFR.

L'implication des participantes dans les services de PTME a été évaluée par une analyse des cascades de soins pour les quatre composants de la PTME. Les cascades de PTME ont été largement utilisées dans la littérature comme outils d'évaluation de la mise en oeuvre et des performances des programmes de PTME dans différents pays.[16-18]Pour le premier composant, c'est-à-dire la prévention primaire des infections au VIH chez les femmes en âge de procréer, nous avons évalué l'utilisation systématique de préservatifs (USP) parmi les TDS séronégatives pour le VIH par type de partenaire sexuel, incluant les clients nouveaux et réguliers, ainsi que les partenaires non payants occasionnels et à long terme. L'USP a été définie comme l'utilisation de préservatifs pendant les 10 plus récents rapports sexuels vaginaux ou anals. Seule l'USP a été prise en compte pour la prévention primaire du VIH parce que la PrEP a été recommandée pour les TDS une fois la collecte des données effectuée pour cette étude.[19]Pour le deuxième composant, c'est-à-dire la prévention des grossesses non désirées chez les femmes vivant avec le VIH, nous avons évalué l'utilisation de méthodes de contraception chez des TDS qui vivaient avec le VIH mais qui n'essayaient pas d'avoir un enfant au moment de l'étude. Le recours à une méthode de contraception a été sous-divisé en deux

catégories : l'utilisation de n'importe quelle forme de contraception et l'utilisation d'une méthode de contraception plus fiable, à long terme, autre qu'une méthode barrière. Pour la première catégorie, une auto-évaluation a été effectuée pour tous les éléments suivants : utilisation de préservatifs pour la planification familiale, pilule contraceptive, dispositif intra-utérin (DIU), contraception hormonale par injection (Depo Provera ou Nuristerate), implant (Norplant ou Jadelle), diaphragme ou cape cervicale et ligature des trompes. Dans les méthodes à long terme, nous avons inclus le DIU, l'implant, les injections et la ligature des trompes. Pour les troisième et quatrième composants, à savoir la prévention de la transmission du VIH des femmes vivant avec le VIH à leurs enfants et la fourniture d'un traitement, de soins et d'un soutien appropriés aux femmes et aux enfants vivant avec le VIH et à leurs familles, les analyses ont été restreintes aux TDS séropositives qui avaient des enfants de moins de 5 ans afin de placer les résultats dans le contexte de la PTME. Nous avons utilisé deux analyses des cascades de PTME pour ces mères et leurs enfants. Les troisième et quatrième composants ont été évalués à l'aide de cascades combinées, l'une basée sur l'implication des mères et l'autre sur les données relatives à leurs enfants. L'analyse de la cascade de soins chez les mères évaluait les informations sur le statut sérologique des mères pour le VIH ainsi que sur leur traitement pendant leur grossesse et dans les années suivant leur grossesse. Bien que le statut sérologique actuel ait été confirmé biologiquement, le statut sérologique pendant la grossesse a été autodéclaré. L'analyse de la cascade de soins chez les enfants était limitée aux enfants nés de mères ayant un diagnostic de VIH connu avant l'étude. La proportion de nourrissons ayant subi un test de dépistage du VIH au moins une fois après la naissance a été évaluée, ainsi que le nombre de bébés ayant subi un nouveau test après l'allaitement, et les transmissions verticales

déclarées par les mères ont été notées. Toutes les analyses ont été effectuées avec le logiciel Stata version 14.2 (StataCorp, College Station, TX).

Résultats

1 069 coupons au total ont été remis dans le cadre de cette étude et 435 femmes se sont présentées sur le site de l'étude. Cependant, parmi ces 1 069 coupons, le nombre de coupons distribués par des TDS participantes à d'autres TDS n'est pas connu. Globalement, 25 femmes ne correspondaient pas aux critères d'éligibilité et 410, dont les neuf « rabatteuses », répondaient aux critères d'éligibilité et ont été recrutées dans l'étude. Le nombre médian de vagues de recrutement EFR était de 6 (EI:4-9) et le maximum atteint était de 16. L'âge médian était de 28 ans (EI:19-51). Parmi ces femmes, 42 % (172/410) étaient célibataires, 84 % (343/410) étaient tombées au moins une fois enceintes et 75 % (307/410) avaient au moins un enfant biologique. La majorité, 70,6 % [(243/343) ; prévalence ajustée sur l'EFR : 66,4 % (IC à 95 % : 58,1-74,8)], des TDS qui avaient été enceintes auparavant ont déclaré avoir eu une grossesse non désirée dans le passé. La prévalence brute du VIH était de 63,7 % (IC à 95 % : 59,0-68,3) ; l'estimation pondérée par l'EFR était de 61,5 % (intervalle de confiance bootstrap à 95 % 54,1-68,0). Les estimations brutes et ajustées sur l'EFR des caractéristiques sociodémographiques et autres caractéristiques sont résumées dans le Tableau 1.

En ce qui concerne l'implication des TDS dans les soins prénatals (SPN) et les services liés à l'accouchement, 95 % (183/192) d'entre elles ont déclaré au moins une visite de SPN et 94 % (181/192) s'étaient vues proposer des services de dépistage du VIH pendant les visites de SPN. 28 % (54/192) des TDS ont été sollicitées par un prestataire pour amener leur partenaire

pendant leur visite de SPN et 2 d'entre elles ont déclaré qu'on leur avait refusé les services de SPN parce que leur partenaire masculin n'était pas venu. Toutes les TDS (100 %) ont déclaré avoir accouché dans un établissement de soins.

En ce qui concerne le travail pendant la grossesse, les femmes travaillant dans l'industrie du sexe avant leur grossesse ont déclaré avoir continué leur travail pendant 5 mois (médiane, EI:4-7) après leur diagnostic de grossesse. En ce qui concerne la reprise du travail après la grossesse, 27 % (38/143) avaient repris leur travail dans l'industrie du sexe dans les trois premiers mois après l'accouchement, et 48 % (69/143) au bout de six mois.

Implication dans la cascade de soins de la PTME

Au moment de l'étude, 5 % (19/410) de toutes les TDS étaient enceintes. Parmi les TDS enceintes, 68 % (13/19) étaient séropositives pour le VIH et 31 % (4/13) de ces dernières suivaient un TAR. Les évaluations de la charge virale chez les femmes enceintes séropositives pour le VIH ont indiqué que la charge virale n'avait été supprimée chez aucune des TDS enceintes vivant avec le VIH.

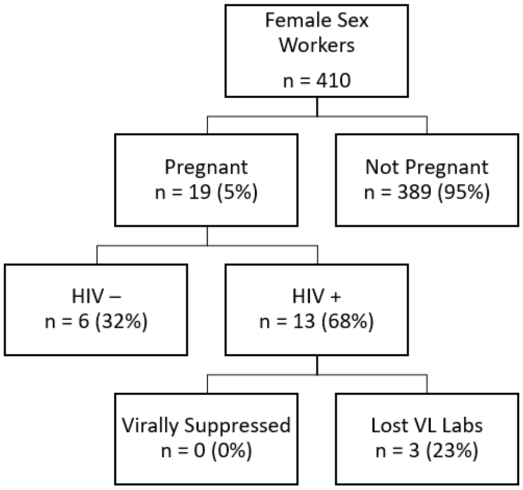

Figure 1. Grossesse et statut sérologique des travailleuses du sexe au moment de l'étude à Port Elizabeth, en Afrique du Sud, entre 2014 et 2015.

(Figure 1) En termes d'implication dans les quatre composants de la PTME, la prévention primaire du VIH chez les femmes en âge de procréer (1er composant) a été évaluée. Au cours de leurs 10 dernières relations sexuelles, 58 % (84/145) des TDS séronégatives pour le VIH avaient systématiquement utilisé des préservatifs avec leurs clients payants, 21 % (4/19) avec leurs partenaires non payants occasionnels et 10 % (8/77) avec leurs partenaires à long terme (Figure 2).

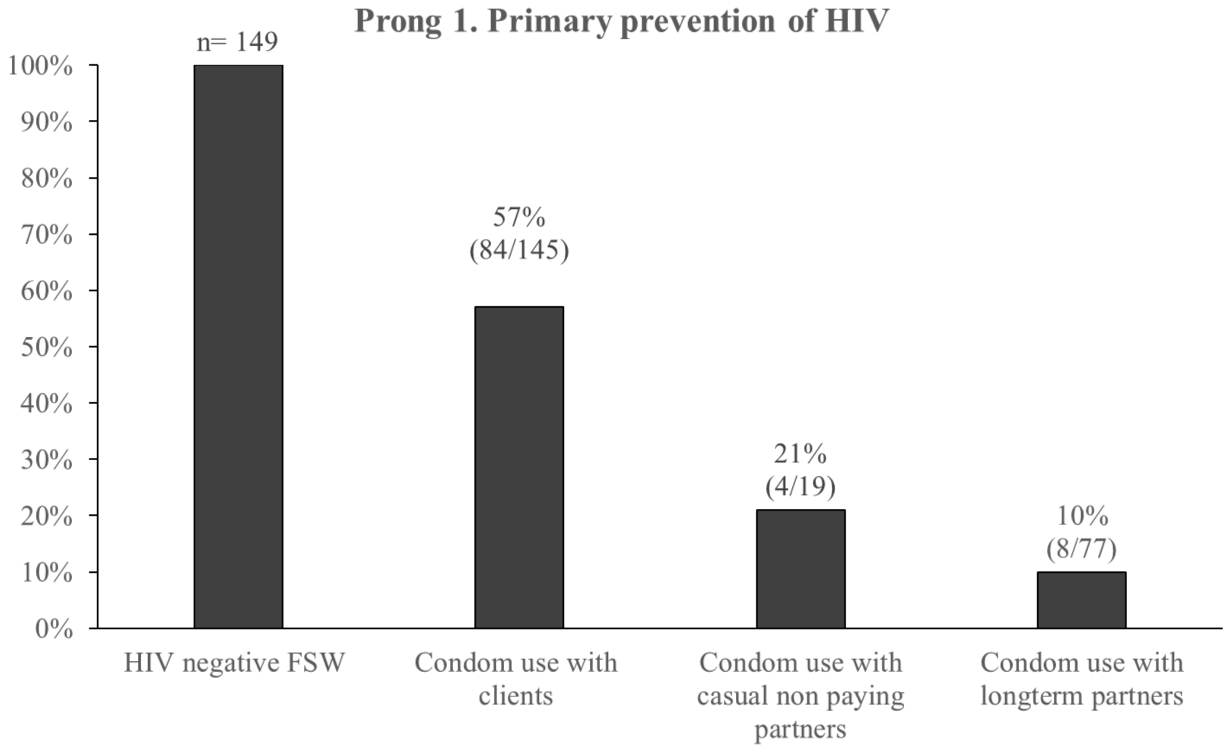

Figure 2. Utilisation systématique de préservatifs au cours des 10 derniers rapports sexuels chez les travailleuses du sexe séronégatives pour le VIH à Port Elizabeth, en Afrique du Sud, entre 2014 et 2015.

Le deuxième composant concernait la prévention des grossesses non désirées chez les femmes vivant avec le VIH. Sur les 261 femmes séropositives de l'étude, 22 essayaient d'avoir un enfant au moment de l'étude et ont été exclues de l'analyse du deuxième composant. Sur les 239 TDS restantes, 91 % (217/239) ont déclaré avoir recours à au moins une méthode de contraception.

Cependant, ce chiffre descendait à 57 % (137/239) pour les méthodes de contraception très efficaces à long terme (Figure 3).

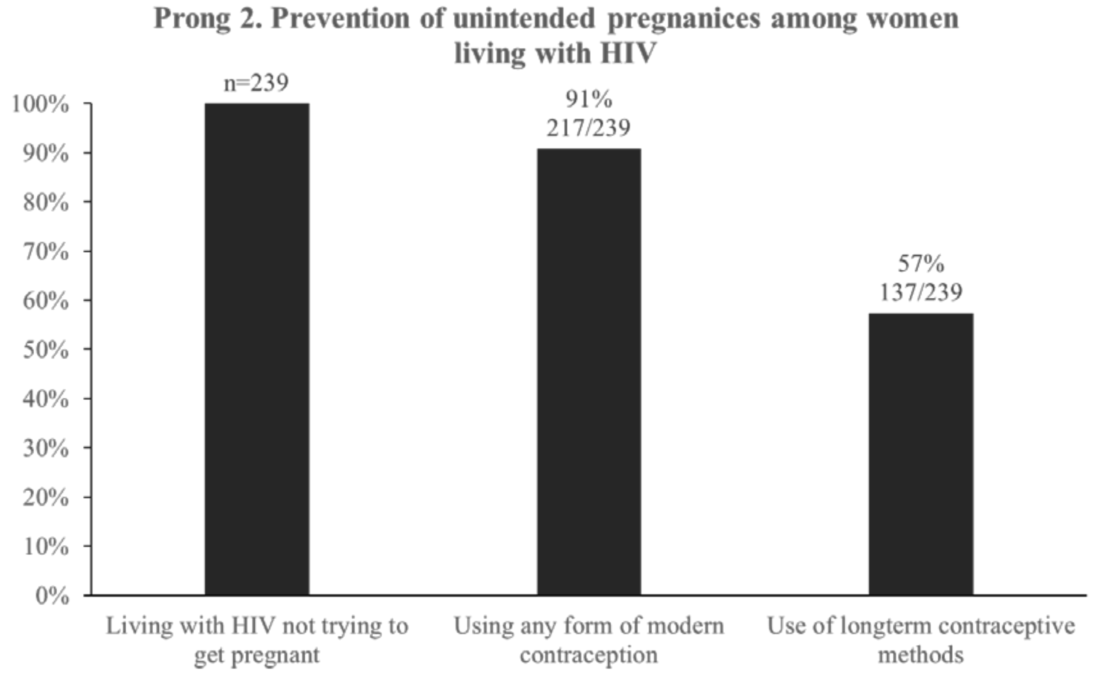

Figure 3. Utilisation d'une méthode de contraception moderne chez les TDS vivant avec le VIH à Port Elizabeth, en Afrique du Sud, entre 2014 et 2015.

En prenant en compte l'implication en termes de traitement des TDS vivant avec le VIH qui avaient des enfants de moins de 5 ans, ainsi que les tests de dépistage des enfants et les résultats de leurs traitements (troisième et quatrième composants), 192 femmes avaient des enfants de moins de 5 ans. Les tests de dépistage du VIH effectués dans le cadre de l'étude ont révélé que 63 % (120/192) des mères étaient séropositives pour le VIH. La majorité des mères (101/120, 84 %) savaient qu'elles étaient séropositives avant l'étude. Parmi les mères séropositives ayant des enfants de moins de 5 ans, 65 % (n=78/120) étaient séropositives au moment de leur dernier accouchement, alors que 35 % (42/120) ont contracté l'infection dans la période postnatale. Parmi celles qui vivaient avec le VIH pendant leur grossesse, 50 % (39/78)

avaient commencé un TAR au moment de leur accouchement. Globalement, parmi toutes les mères vivant avec le VIH ayant des enfants de moins de 5 ans, 45 % (45/101) étaient sous TAR (Figure 4).

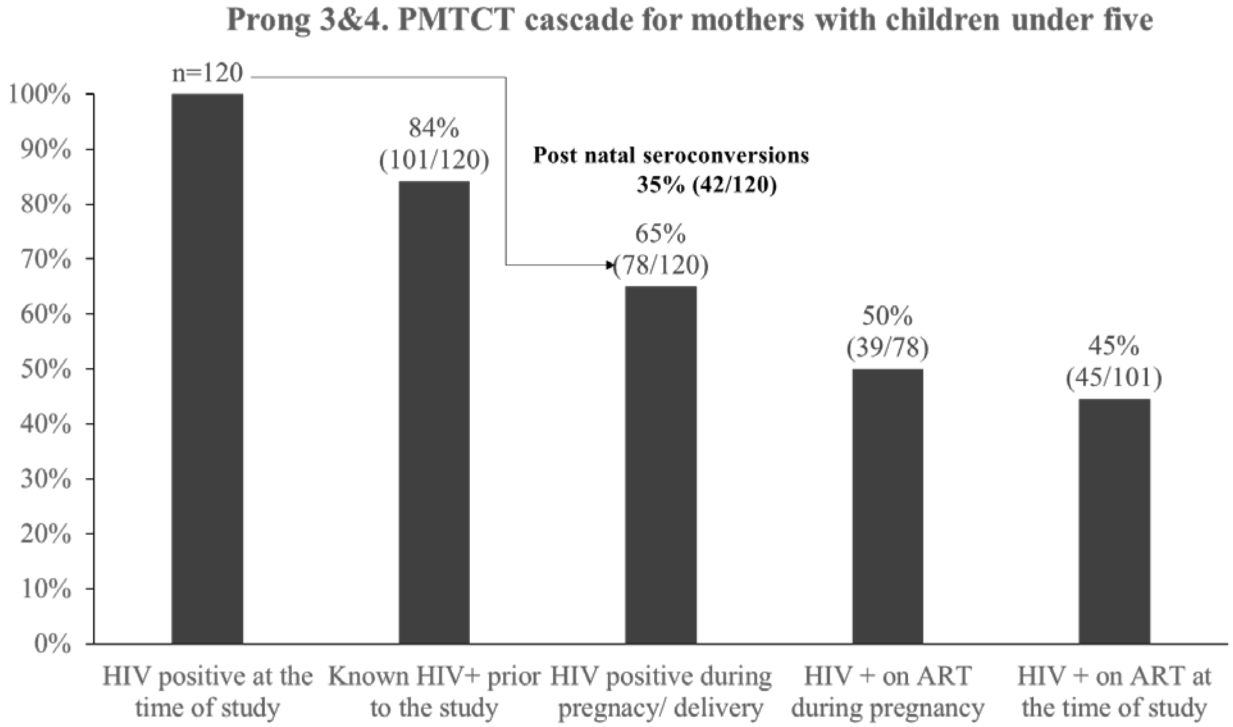

Figure 4. Cascade de soins de PTME pour les TDS séropositives pour le VIH ayant des enfants de moins de 5 ans à Port Elizabeth, en Afrique du Sud, entre 2014 et 2015.

Sur les 101 femmes dont on savait qu'elles vivaient avec le VIH avant l'étude, 85 % (86/101) avaient fait passer un test de dépistage du VIH à leurs enfants au moins une fois après leur naissance. La majorité des mères (85 %, n=86) avaient allaité, et 36 % (31/86) de ces dernières avaient refait passer un test de dépistage à leurs enfants après la fin de l'allaitement. Globalement, 12 % (12/101) des mères précédemment diagnostiquées ont déclaré avoir au moins un enfant de moins de 5 ans vivant avec le VIH. (Figure 5)

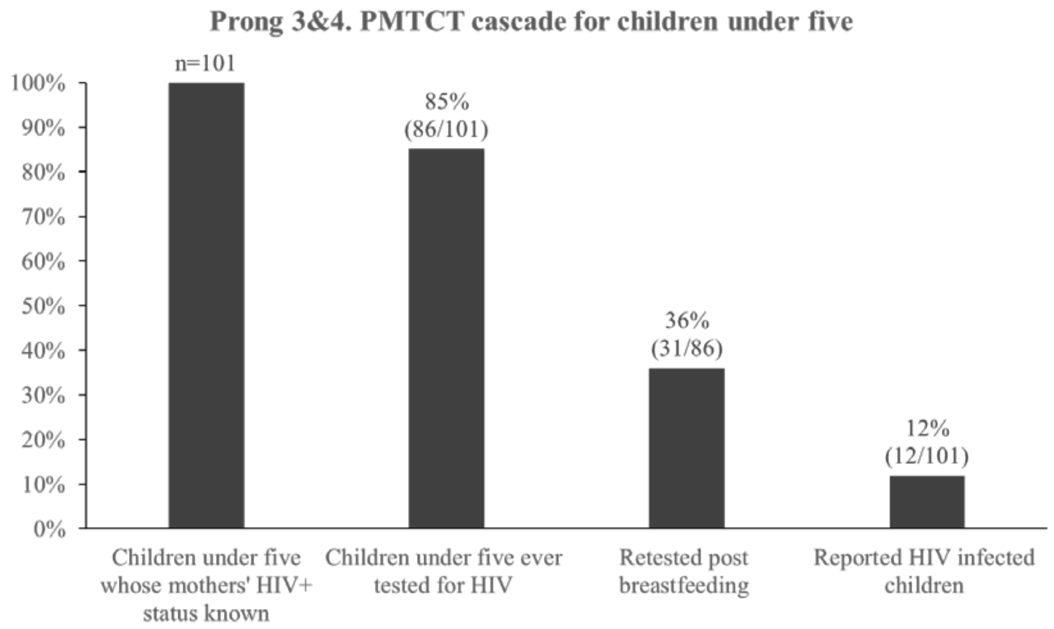

Figure 5. Cascade de soins de PTME pour les enfants de moins de 5 ans nés avec le VIH à Port Elizabeth, en Afrique du Sud, entre 2014 et 2015.

Discussion

Une fois regroupées, ces données fournissent la preuve de l'implication sous-optimale des TDS dans le continuum de soins de PTME, révélée par des manquements dans chacun des quatre composants de l'approche PTME recommandée par l'OMS. Les manquements remarquables observés comprenaient : l'utilisation non systématique de préservatifs avec les clients et les partenaires non payants chez les TDS séronégatives, un faible recours à des méthodes de contraception plus fiables chez les TDS séropositives, une couverture TAR insuffisante chez les TDS mères vivant avec le VIH et un faible taux de tests de dépistage des enfants après la période d'allaitement. Les risques de transmission verticale ont été davantage mis en évidence par les infections signalées par les mères. En effet, 12 % ont déclaré avoir au moins un enfant vivant avec le VIH.

Les taux élevés de contraction du VIH par les mères dans les mois et les années suivant un accouchement (35 % des infections observées étant survenues chez les nouvelles mères) soulignent encore la nécessité de renforcer les efforts de prévention du VIH pendant toute la période d'allaitement et au-delà. La protection de la mère et de l'enfant pendant cette période est primordiale car près de 50 % des TDS interrogées reprenaient leur travail dans l'industrie du sexe dans les six mois suivant l'accouchement. Le risque élevé de contraction du VIH dans les années suivant l'accouchement souligne la nécessité d'apporter une plus grande attention au premier composant de la PTME et de renforcer les efforts de prévention primaire pour les nouvelles mères, en prônant notamment l'utilisation systématique de préservatifs mais également la prophylaxie préexposition (PrEP). Par ailleurs, les directives de PTME en Afrique du Sud préconisent des tests de dépistage du VIH répétés pour les femmes séronégatives pendant la grossesse et la période post-partum.[4] De plus, étant donné les fréquentes séroconversions postnatales chez les TDS et les risques élevés de TME associés à la séroconversion pendant la grossesse[20], il peut être particulièrement important de prôner des tests répétés chez les TDS enceintes et allaitantes afin de permettre une détection précoce de nouvelles infections par le VIH et une mise en place rapide des traitements.[21] Enfin, on pourrait également encourager le recours à la PrEP pour les TDS enceintes et allaitantes, d'autant plus que les résultats sont favorables jusqu'à aujourd'hui en matière de sécurité et qu'il s'agit d'une recommandation actuelle de l'OMS.[22-24]

Même si l'utilisation d'une méthode de contraception était élevée chez les TDS vivant avec le VIH, seulement un peu plus de la moitié d'entre elles ont déclaré avoir recours à des méthodes de contraception à long terme, autres que les méthodes barrières. Ce point est primordial au vu

de la proportion élevée de grossesses non désirées signalées chez les TDS dans cette étude et dans des études précédentes. [25-27] Des tendances similaires ont été observées chez des TDS en Inde, au Kenya, au Mozambique et en Afrique du Sud.[28] Des efforts supplémentaires pour augmenter le recours à une méthode de contraception plus fiable sont nécessaires en raison des taux élevés de grossesses non désirées et des répercussions néfastes associées, notamment des consultations tardives en SPN et un faible poids de naissance des enfants. [29-31] En outre, les grossesses non désirées chez les TDS entraînent également des conséquences sociales et économiques, notamment une diminution de leur capacité de travail et une perte de revenus.[26]

L'un des principaux aspects de ce travail est de comprendre les risques de transmission verticale chez les TDS vivant avec le VIH. La prise d'un TAR pendant la grossesse et la période post-partum était un manquement crucial observé chez les TDS mères. 50 % seulement des TDS vivant avec le VIH avaient démarré un TAR pendant leur grossesse et moins de la moitié des TDS ayant des enfants de moins de 5 ans suivaient un TAR au moment de l'étude. En comparaison, 90 % de toutes les femmes enceintes vivant avec le VIH en Afrique du Sud suivent un TAR.[3] La faible couverture en TAR observée chez les TDS a également été observée dans d'autres études. Ces données montrent que les services de PTME classiques ne sont pas adaptés aux TDS. En réalité, pratiquement toutes les TDS ayant des enfants de moins de 5 ans bénéficiaient de services de SPN et avaient accès à des tests de dépistage du VIH pendent leur grossesse mais n'avaient pas accès aux soins et aux traitements. Cela montre que, malgré une augmentation réussie des services de PTME en Afrique du Sud, des disparités sanitaires existent en termes de couverture PTME chez les TDS. Cela montre également que les programmes

efficaces pour les autres femmes en âge de procréer ne fonctionnement pas nécessairement pour les TDS en raison principalement de facteurs structurels propres aux TDS qui réduisent leur capacité à accéder aux services de prévention du VIH et aux traitements. [11] Les pratiques discriminatoires peuvent également jouer un rôle. En effet, d'autres études ont révélé que l'insistance à ce que les partenaires masculins soient présents lors des SPN éloignaient les femmes des soins.[32] Par ailleurs, chez les quelques TDS se trouvant enceintes et sous TAR au moment de l'étude, la charge virale n'avait été supprimée pour aucune d'entre elles, ce qui signifie que même celles qui étaient sous traitement pouvaient encore présenter un risque élevé de TME.[33, 34] Il est donc nécessaire de mettre en place des programmes spécifiques adaptés aux TDS mères pour améliorer la couverture en TAR et la suppression de la charge virale.

Les risques de transmission chez les TDS peuvent être supérieurs à la moyenne nationale car 12 % des TDS dont on sait qu'elles vivaient avec le VIH avant l'étude ont déclaré avoir au moins un enfant vivant avec le VIH. Par ailleurs, le taux de recours à des tests de dépistage supplémentaires des enfants après l'allaitement n'étant que de 30 %, il existe probablement d'autres cas de TME non diagnostiqués.

La stratégie nationale menée par les pairs pour les TDS en Afrique du Sud offre une opportunité unique de pallier aux manquements dans la cascade de soins PTME pour les TDS.[19]Les pairs éducateurs enrôlés dans les équipes communautaires de proximité et les centres d'accueil centraux peuvent aider les responsables du programme à identifier les TDS qui sont enceintes et à les accompagner pour leurs premières consultations en SPN, soutenir leur observance du TAR, les encourager à faire passer des tests de dépistage à leurs enfants, repérer les couples

mère/enfant ayant échappé au suivi et soutenir les mères pendant le traitement des enfants infectés.

Néanmoins, cette étude a des limites. Tout d'abord, l'analyse s'appuie sur des informations autodéclarées pour l'utilisation du TAR, le diagnostic de VIH antérieur et le statut sérologique des enfants, informations qui sont toutes sujettes à des biais de rappel et de désirabilité sociale. Toutefois, la corrélation élevée entre le diagnostic de VIH autodéclaré et les résultats des tests augmente la confiance dans la précision de l'autoévaluation. En outre, il y a peu de raisons de croire que le nombre d'enfants infectés par le VIH aurait été surévalué. Par contre, il aurait pu être sous-évalué, d'où l'importance d'intégrer des tests de dépistage du VIH chez les enfants de TDS dans les données de la recherche et du programme. Le fait d'avoir restreint les analyses aux femmes ayant des enfants de moins de 5 ans a permis de placer les résultats dans le contexte de la PTME mais a réduit la taille de l'échantillon, empêchant de réaliser des analyses plus complexes. Enfin, la conception transversale a limité notre capacité à évaluer les relations temporelles, en particulier pour la transmission verticale. Malgré ces limites, des risques clairs de TME ont été identifiés dans le groupe des mères.

Conclusion

Cette étude est l'une des seules à évaluer le niveau d'implication dans la cascade de soins de PTME et les risques de transmission verticale chez les TDS en Afrique du Sud et en Afrique subsaharienne. Les résultats montrent que, malgré la mise en oeuvre réussie de programmes de PTME et la réduction spectaculaire de la transmission verticale du VIH en Afrique du Sud, les TDS peuvent avoir un moins bon accès aux services de PTME par rapport aux autres femmes en

âge de procréer. Les TDS présentent des risques élevés de contraction du VIH, elles ont de nombreux besoins non pris en charge en matière de planification familiale, un faible taux de prise de TAR pendant leurs grossesses et dans la période postnatale, et elles présentent parfois des taux de TME plus élevés que la moyenne nationale. Il est donc primordial de renforcer les programmes de PTME spécifiques aux TDS afin d'améliorer leur santé et celle de leurs enfants.

Liste des abréviations :

SPN : Soins prénatals
TAR : Traitement antirétroviral
USP : Utilisation systématique de préservatifs
TDS : Travailleuses du sexe
VIH : Virus de l'immunodéficience humaine
EI : Écart interquartile
DIU : Dispositif intra-utérin
TME : Transmission mère-enfant du VIH
NMBM : Nelson Mandela Bay Metropolitan Municipality (métropole de la baie Nelson Mandela)
PTME : Prévention de la transmission mère-enfant du VIH
PrEP : Prophylaxie préexposition
EFR : Échantillonnage fondé sur les répondants
ASS : Afrique subsaharienne
SDSR : Santé et droits sexuels et reproductifs
IST : Infection sexuellement transmissible
OMS : Organisation mondiale de la Santé

Concernant ce supplément

Cet article a été publié comme partie de l'ouvrage de *BMC Public Health*, Volume 19 Supplement 1, 2019: Effective Integration of Sexual Reproductive Health and HIV Prevention, Treatment, and Care Services across sub-Saharan Africa: Where is the evidence for program implementation?
Le supplément a été publié dans le cadre d'une collaboration entre *Reproductive Health* et *BMC Public Health.* L'intégralité du contenu, avec les versions en français portugais et anglais, est disponible en ligne :
https://bmcpublichealth.biomedcentral.com/articles/supplements/volume-19-supplement-1
et
https://reproductive-health-journal.biomedcentral.com/articles/supplements/volume-16-supplement-1

Déclaration

Approbation éthique et accord de participation

L'étude a été approuvée par les comités d'examens institutionnels de la Johns Hopkins Bloomberg School of Public Health et du Human Sciences Research Council of South Africa. Tous les participants ont rempli un formulaire de consentement éclairé pour participer à l'étude.

Accord de publication

Non applicable

Disponibilité des données et matériels

Les données appartiennent aux établissements partenaires. Les demandes d'utilisation des données doivent être envoyées à Sheree Schwartz à l'adresse : sschwartz@jhu.edu.

Conflits d'intérêts

Les auteurs déclarent ne pas avoir de conflits d'intérêts.

Financement

Cette étude a été financée par la fondation MAC AIDS Fund. Elle a été mise en oeuvre par le Human Sciences for Research Council (HSRC) et la TB/HIV Care Association (THCA), en collaboration avec l'université Johns Hopkins. Cette recherche a été facilitée par l'infrastructure et les ressources fournies par le Johns Hopkins University Center for AIDS Research, un programme financé par les National Institutes of Health (1P30AI094189), qui est soutenu par

les instituts et centres participants et co-financeurs des NIH suivants : NIAID, NCI, NICHD, NHLBI, NIDA, NIMH, NIA, FIC, NIGMS, NIDDK et OAR.

Le supplément de la revue est rendu possible grâce au soutien généreux du peuple américain via la United States Agency for International Development (USAID) en partenariat avec le Fonds des Nations unies pour la population (FNUAP) et le Programme commun des Nations Unies sur le VIH/SIDA (ONUSIDA).

Les opinions exprimées dans la présente publication sont celles des auteurs et ne reflètent pas nécessairement les politiques officielles de l'USAID, du FNUAP ou de l'ONUSIDA, la mention des dénominations de ministères ou d'organismes n'implique pas non plus l'aval du gouvernement américain, du FNUAP ou de l'ONUSIDA.

Contributions des auteurs

ShS, SB, NP-M, ZK, AL et MM ont conçu l'étude ; ZK, MM, ShS, AL, NP-M, SK, HH et SB ont tous soutenu la mise en oeuvre de l'étude. JOTR, ShS, AR et SK ont supervisé les analyses. JOTR, ShS et SB ont rédigé le manuscrit et tous les auteurs ont contribué à son contenu. Tous les auteurs ont lu et approuvé le manuscrit final.

Remerciements

Nous remercions les femmes ayant participé à l'étude pour leur temps et pour avoir partagé leurs expériences avec nous. Nous remercions également toutes les institutions et les personnes impliquées dans la conception et la mise en oeuvre de cette étude. Les auteurs

remercient également la société Johnson & Johnson pour ses dons de fournitures aux femmes qui ont participé à cette étude.

Références

1. UNAIDS: **Global Plan Towards the Elimination of New Infections in Children by 2015 and Keeping Mothers Alive, 2011–2015**. In. Geneva, Switzerland; 2011.
2. World Health O: **PMTCT Strategic Vision 2010–2015: Preventing Mother-to-Child Transmission of HIV to Reach the UNGASS and Millennium Development Goals**. In. Geneva, Switzerland; 2010.
3. UNAIDS: **On Fast-Track to an AIDS Free- Generation** In. Geneva, Switzerland: UNAIDS 2016.
4. Health Do: **National Consolidated guidelines for the Prevention of Mother-To-child transmission of HIV (PMTCT) and the management of HIV in Children, Adolescents and Adults**. In.: National Department of Health - South Africa; 2014.
5. UNAIDS: **Prevention gap report**. In.; 2016.
6. Schwartz SR, Baral S: **Fertility-related research needs among women at the margins**. *Reproductive Health Matters* 2015, **23**(45):30-46.
7. Baral S, Beyrer C, Muessig K, Poteat T, Wirtz AL, Decker MR, Sherman SG, Kerrigan D: **Burden of HIV among female sex workers in low-income and middle-income countries: A systematic review and meta-analysis**. *The Lancet Infectious Diseases* 2012, **12**(7):538-549.
8. Papworth E, Ceesay N, An L, Thiam-Niangoin M, Ky-Zerbo O, Holland C, Dramé FM, Grosso A, Diouf D, Baral SD: **Epidemiology of HIV among female sex workers, their clients, men who have sex with men and people who inject drugs in West and Central Africa**. In., vol. 16 Suppl 3: The International AIDS Society; 2013: 18751-18751.
9. Ngugi EN, Roth E, Mastin T, Nderitu MG, Yasmin S: **Female sex workers in Africa: epidemiology overview, data gaps, ways forward**. *SAHARA J : journal of Social Aspects of HIV/AIDS Research Alliance / SAHARA , Human Sciences Research Council* 2012, **9**(3):148-153.
10. Shannon K, Goldenberg SM, Deering KN, Strathdee SA: **HIV infection among female sex workers in concentrated and high prevalence epidemics: why a structural determinants framework is needed**. *Current opinion in HIV and AIDS* 2014, **9**(2):174-182.
11. Shannon K, Strathdee SA, Goldenberg SM, Duff P, Mwangi P, Rusakova M, Reza-Paul S, Lau J, Deering K, Pickles MR *et al*: **Global epidemiology of HIV among female sex workers: Influence of structural determinants**. *The Lancet* 2015, **385**(9962):55-71.
12. Heckathorn DD, Wangroongsarb P, Thwing J, Eliades J, Satimai W, Delacollette C, Kaewkungwal J, Zhou G, Sirichaisinthop J, Sattabongkot J *et al*: **Respondent-Driven Sampling: A New Approach to the Study of Hidden Populations**. *Social Problems* 1997, **44**(2):174-199.

13. Schwartz S, Lambert A, Phaswana-Mafuya N, Kose Z, McIngana M, Holland C, Ketende S, Yah C, Sweitzer S, Hausler H *et al*: **Engagement in the HIV care cascade and barriers to antiretroviral therapy uptake among female sex workers in Port Elizabeth, South Africa: findings from a respondent-driven sampling study**. *Sexually Transmitted Infections* 2016:sextrans-2016-052773.
14. Rao A, Baral S, Phaswana-Mafuya N, Lambert A, Kose Z, McIngana M, Holland C, Ketende S, Schwartz S: **Pregnancy Intentions and Safer Pregnancy Knowledge Among Female Sex Workers in Port Elizabeth, South Africa**. *Obstet Gynecol* 2016, **128**(1):15-21.
15. Health RoSANDo: **National HIV Counselling and Testing (HCT) Policy Guidelines: South Africa**. In.; 2010.
16. Hamilton E, Bossiky B, Ditekemena J, Esiru G, Fwamba F, Goga AE, Kieffer MP, Tsague LD, van de Ven R, Wafula R *et al*: **Using the PMTCT Cascade to Accelerate Achievement of the Global Plan Goals**. *JAIDS Journal of Acquired Immune Deficiency Syndromes* 2017, **75**:S27-S35.
17. Hofer CB, Egger M, Davies M-A, Frota ACC, de Oliveira RH, Abreu TF, Araújo LE, Witthlin BB, Carvalho AW, Cordeiro JR *et al*: **The cascade of care to prevent mother-to-child transmission in Rio de Janeiro, Brazil, 1996-2013: improving but still some way to go**. *Tropical Medicine & International Health* 2017.
18. Dionne-Odom J, Welty TK, Westfall AO, Chi BH, Ekouevi DK, Kasaro M, Tih PM, Tita ATN: **Factors associated with PMTCT cascade completion in four african countries**. *AIDS Research and Treatment* 2016, **2016**.
19. SANAC: **The South African National Sex Worker HIV Plan, 2016 - 2019**. In.: South African National Aids Council (SANAC); 2016.
20. Dinh TH, Delaney KP, Goga A, Jackson D, Lombard C, Woldesenbet S, Mogashoa M, Pillay Y, Shaffer N: **Impact of Maternal HIV Seroconversion during Pregnancy on Early Mother to Child Transmission of HIV (MTCT) Measured at 4-8 Weeks Postpartum in South Africa 2011-2012: A National Population-Based Evaluation**. *PLoS One* 2015, **10**(5):e0125525.
21. Bispo S, Chikhungu L, Rollins N, Siegfried N, Newell ML: **Postnatal HIV transmission in breastfed infants of HIV-infected women on ART: A systematic review and meta-analysis**. In., vol. 20; 2017.
22. Mugwanya KK, John-Stewart G, Baeten J: **Safety of oral tenofovir disoproxil fumarate-based HIV pre-exposure prophylaxis use in lactating HIV-uninfected women**. *Expert Opin Drug Saf* 2017, **16**(7):867-871.
23. Mugwanya KK, Hendrix CW, Mugo NR, Marzinke M, Katabira ET, Ngure K, Semiyaga NB, John-Stewart G, Muwonge TR, Muthuri G *et al*: **Pre-exposure Prophylaxis Use by Breastfeeding HIV-Uninfected Women: A Prospective Short-Term Study of Antiretroviral Excretion in Breast Milk and Infant Absorption**. *PLoS Med* 2016, **13**(9):e1002132.
24. WHO: **Preventing HIV during Pregnancy and Breastfeeding in the context of PREP**. In. Geneva; 2017.
25. S SS, Papworth E, Thiam-Niangoin M, Abo K, Drame F, Diouf D, Bamba A, Ezouatchi R, Tety J, Grover E *et al*: **An Urgent Need for Integration of Family Planning Services Into HIV Care: The High Burden of Unplanned Pregnancy, Termination of Pregnancy, and**

Limited Contraception Use Among Female Sex Workers in Cote d'Ivoire. TT. *Journal of Acquired Immune Deficiency Syndromes* 2015, **68 Suppl 2**:S91-S98.

26. Luchters S, Bosire W, Feng A, Richter ML, King'ola N, Ampt F, Temmerman M, Chersich MF: **"A Baby Was an Added Burden": Predictors and Consequences of Unintended Pregnancies for Female Sex Workers in Mombasa, Kenya: A Mixed-Methods Study**. *PLoS One* 2016, **11**(9):e0162871.
27. Chanda MM, Ortblad KF, Mwale M, Chongo S, Kanchele C, Kamungoma N, Barresi LG, Harling G, Barnighausen T, Oldenburg CE: **Contraceptive use and unplanned pregnancy among female sex workers in Zambia**. *Contraception* 2017, **96**(3):196-202.
28. Lafort Y, Greener R, Roy A, Greener L, Ombidi W, Lessitala F, Skordis-Worrall J, Beksinska M, Gichangi P, Reza-Paul S *et al*: **Sexual and reproductive health services utilization by female sex workers is context-specific: results from a cross-sectional survey in India, Kenya, Mozambique and South Africa**. *Reprod Health* 2017, **14**(1):13.
29. Kost K, Lindberg L: **Pregnancy intentions, maternal behaviors, and infant health: investigating relationships with new measures and propensity score analysis**. *Demography* 2015, **52**(1):83-111.
30. Hall JA, Benton L, Copas A, Stephenson J: **Pregnancy Intention and Pregnancy Outcome: Systematic Review and Meta-Analysis**. *Matern Child Health J* 2017, **21**(3):670-704.
31. Lindberg L, Maddow-Zimet I, Kost K, Lincoln A: **Pregnancy intentions and maternal and child health: an analysis of longitudinal data in Oklahoma**. *Matern Child Health J* 2015, **19**(5):1087-1096.
32. Beckham SW, Shembilu CR, Brahmbhatt H, Winch PJ, Beyrer C, Kerrigan DL: **Female Sex Workers' Experiences with Intended Pregnancy and Antenatal Care Services in Southern Tanzania**. *Studies in Family Planning* 2015, **46**(1):55-71.
33. Mandelbrot L, Tubiana R, Le Chenadec J, Dollfus C, Faye A, Pannier E, Matheron S, Khuong M-A, Garrait V, Reliquet V *et al*: **No Perinatal HIV-1 Transmission From Women With Effective Antiretroviral Therapy Starting Before Conception**. *Clinical Infectious Diseases* 2015, **61**(11):1715-1725.
34. Myer L, Phillips TK, Hsiao NY, Zerbe A, Petro G, Bekker LG, McIntyre JA, Abrams EJ: **Plasma viraemia in HIV-positive pregnant women entering antenatal care in South Africa**. *J Int AIDS Soc* 2015, **18**:20045.

Tableau 1. Caractéristiques sociodémographiques et SDSR des travailleuses du sexe participant à l'étude à Port Elizabeth, en Afrique du Sud, entre 2014 et 2015

**Caractéristique**	**N**	**% brut**	**% ajusté sur l'EFR [IC 95 %]**
**Âge**			
18-24	122	29,8	38,2 (30,1-46,3)
25-34	205	50	44,0 (37,0-51,0)
> 35	83	20,2	17,8 (12,2-23,5)
**Race**			
Noire africaine	337	83,2	74,5 (60,6-88,4)
Autre	68	16,8	25,5 (11,6-39,4)
**Éducation**			
Niveau collège	87	21,2	22,9 (17,2-28,5)
Secondaire partiel	158	38,5	36,5 (30,5-42,5)
Niveau secondaire ou plus	165	40,3	40,6 (33,9-47,3)
**Situation relationnelle**			
Célibataire	172	42,0	39,7 (32,9-46,4)
En relation avec un partenaire stable	238	58,0	60,3 (53,6,-67,1)
**Antécédents de grossesse**			
N'a jamais été enceinte	67	16,3	15,9 (11,7-20,3)
A été au moins une fois enceinte	343	83,7	84,1 (79,7-88,3)
**Antécédents de grossesse non désirée chez les TDS ayant des antécédents de grossesse**			
N'a jamais eu de grossesse non	100	29,3	33,5 (25,2,-41,9)

désirée			
A eu au moins une grossesse non désirée	243	70,6	66,4 (58,1-74,8)
**Enfants biologiques**			
N'a pas d'enfant	103	25,1	24,8 (19,3-30,4)
A au moins un enfant	307	74,9	75,2 (69,6-80,7)
**Revenu moyen par semaine**			
Moins de 500 ZAR	151	36,8	37,1 (30,1-43,3)
Plus de 500 ZAR	259	63,2	62,9 (56,7-69,3)
**Activité**			
Travail dans l'industrie du sexe uniquement	391	95,4	93,7 (89,7-97,7)
Activité supplémentaire	19	4,6	6,3 (2,3-10,2)
**Symptômes d'IST au cours de l'année précédente**			
Non	255	62,2	63,4 (56,4,-70,5)
Oui	155	37,8	36,6 (30,1-43,2)
**Infection par le VIH**			
Séronégative	149	36,3	38,5 (31,2-45,8)
Séropositive	261	63,7	61,5 (54,2-68,8)
