# Supplementary material for: Evaluating the vertical HIV transmission risks among South African female sex workers; have we forgotten PMTCT in their HIV programming?
Source: BMC Public Health. 2019 May 29;19(Suppl 1):605. doi: 10.1186/s12889-019-6811-4 (PMC6538543; doi:10.1186/s12889-019-6811-4)
Supplement: Supplementary file 2 — Translation of the abstract of this article into Portuguese. (PDF 107 kb) [file 12889_2019_6811_MOESM2_ESM.pdf]

BMC Public Health 2019, 19 (Suppl 1) [translated from the original publication]

Avaliar os riscos de transmissão vertical do HIV e a PTMF em cadeia entre as trabalhadoras do sexo na África do Sul. Teremos esquecido a PTMF na programação do VIH para este grupo?

Autores: Jean Olivier Twahirwa Rwema[1*] , Stefan Baral,[1,] Sosthenes Ketende[1,] Nancy Phaswana-Mafuya[3,4] , Andrew Lambert[2] , Zamakayise Khose[3] , Mfezi Mcingana[5] , Amrita Rao[1], Harry Hausler[2] , Sheree Schwartz[1]

Afiliações:

1. Department of Epidemiology, Center for Public Health and Human Rights, Johns Hopkins Bloomberg School of Public Health, Baltimore, Maryland, USA
2. The TB/HIV Care Association, Cape Town, South Africa
3. The Human Sciences Research Council, Port Elizabeth, South Africa
4. Nelson Mandela Metropolitan University, Port Elizabeth, South Africa
5. The TB/HIV Care Association, Port Elizabeth, South Africa

***Correspondência:** Jean Olivier Twahirwa Rwema, MD, MPH, Department of Epidemiology, Key Populations Program, Center for Public Health and Human Rights, Johns Hopkins Bloomberg School of Public Health, 615 N Wolfe Street E 7133 Baltimore, MD 21205 (email: jtwahir1@jhu.edu)

Endereços de e-mail dos autores:
Jean Olivier Twahirwa Rwema: jtwahir1@jhmi.edu
Stefan Baral: sbaral@jhu.edu
Sosthenes Ketende: sketende@jhu.edu
Nancy Phaswana-Mafuya: nphaswanamafuya@hsrc.ac.za
Andrew Lambert: lambertandy@gmail.com
Zamakayise Khose: zkose@hsrc.ac.za
Mfezi Mcingana: mfezi@tbhivcare.org
Amrita Rao: arao24@jhu.edu
Harry Hausler: hhausler@tbhivcare.org
Sheree Schwartz: sschwartz@jhu.edu

Resumo

Introdução: As trabalhadoras do sexo têm um fardo mais pesado em termos do VIH em comparação com outras mulheres no período reprodutivo e apresentam uma elevada incidência de gravidezes. Contudo, existem dados limitados sobre a transmissão mãe-filho do VIH no contexto do trabalho sexual. Este estudo avaliou a adoção dos serviços de prevenção da transmissão mãe-filho (PTMF) para compreender os riscos da transmissão vertical do VIH entre as trabalhadoras do sexo na África do Sul.

Métodos: Foram selecionadas trabalhadoras do sexo com idade superior ou igual a 18 anos para um estudo transversal, utilizando a amostragem orientada por respondentes (RDS) entre

outubro 2014 e abril de 2015 em Port Elizabeth, na África do Sul. Um inquérito conduzido por um entrevistador recolheu informações sobre dados demográficos, histórias de saúde reprodutiva e cuidados do VIH, incluindo a utilização de cuidados de PTMF e TAR. Os testes de VIH e gravidez foram avaliados a nível biológico. Esta análise caracteriza a participação das trabalhadoras do sexo na prevenção do VIH e os tratamentos em cadeia de quatro vertentes da PTMF.

Resultados: No geral, o estudo contou com a participação de 410 trabalhadoras do sexo. O valor de prevalência do VIH da RDS foi de 61,5% (intervalo de confiança de 95% com uma distribuição bootstrap de 54,1-68,0). Uma avaliação exaustiva das quatro vertentes da PTMF apresentou disparidades em cadeia para cada uma das vertentes. Nas vertentes 1 e 2, foi observada uma disparidade de 42% que consiste na utilização de preservativo com clientes entre trabalhadoras do sexo seronegativas e uma disparidade de 43% que consiste na utilização de um método contracetivo de elevada eficiência a longo prazo entre trabalhadoras do sexo seropositivas. As análises das vertentes 3 e 4 dizem respeito a 192 mulheres que tinham filhos com menos de cinco anos; nesta amostragem, 101/192 tinham conhecimento do seu diagnóstico de VIH antes do estudo, das quais 85% (86/101) realizaram testes de despiste do VIH para os seus filhos após o nascimento, contudo, apenas 36% (31/86) das mulheres que amamentavam os seus filhos submeteram-nos novamente ao teste após o período de amamentação. Uma proporção substancial (35%, 42/120) de todas as mulheres seropositivas que tinham filhos com menos de cinco anos era seronegativa no último parto e tornou-se seropositiva após o parto. Menos de metade (45%) das mães que tinham filhos com menos de

cinco anos (45/101) estava a receber TAR e 12% (12/101) indicava ter, pelo menos, um filho com menos de cinco anos a viver com o VIH.

Conclusão: Estas conclusões apresentam lacunas significativas na participação em PTMF em cadeia para as trabalhadoras do sexo, tal como é demonstrado pela fraca adoção da prevenção do VIH e tratamento nos períodos pré/pós-parto, bem como pela prevenção insuficiente de gravidez indesejada entre as trabalhadoras do sexo a viver com o VIH. Estas lacunas resultam em riscos elevados de transmissão vertical entre as trabalhadoras do sexo e mostram a necessidade de existirem serviços de PTMF integrados nos programas para as trabalhadoras do sexo.

Palavras-chave: PTMF, transmissão vertical, trabalhadoras do sexo, África do Sul

Sobre este suplemento

Este resumo foi publicado como parte da revista científica *BMC Public Health*, Volume 19, Suplemento 1, 2019: Integração Eficaz dos Serviços de Saúde Sexual e Reprodutiva e de Prevenção, Cuidados e Tratamento do VIH na África Subsariana: Onde estão as provas da implementação do programa?
O suplemento foi publicado como uma colaboração entre as revistas científicas *Reproductive Health* e *BMC Public Health.* O conteúdo integral, incluindo as versões em francês, português e inglês, estão disponíveis online:
https://bmcpublichealth.biomedcentral.com/articles/supplements/volume-19-supplement-1 e https://reproductive-health-journal.biomedcentral.com/articles/supplements/volume-16-supplement-1
